# Underwater TDOA Acoustical Location Based on Majorization-Minimization Optimization

**DOI:** 10.3390/s20164457

**Published:** 2020-08-10

**Authors:** Shuangshuang Li, Haixin Sun, Hamada Esmaiel

**Affiliations:** 1 Key Laboratory of Underwater Acoustic Communication and Marine Information Technology, Ministry of Education, School of Informatics, Xiamen University, Xiamen 361005, China; shuangshuangli@stu.xmu.edu.cn (S.L.); h.esmaiel@aswu.edu.eg (H.E.); 2 Department of Information and Communication, School of Informatics, Xiamen University, Xiamen 361005, China; 3 Electrical Engineering Department, Faculty of Engineering, Aswan University, Aswan 81542, Egypt

**Keywords:** majorization-minimization, time difference of arrival, underwater acoustic localization, underwater acoustic sensor networks

## Abstract

Underwater acoustic localization is a useful technique applied to any military and civilian applications. Among the range-based underwater acoustic localization methods, the time difference of arrival (TDOA) has received much attention because it is easy to implement and relatively less affected by the underwater environment. This paper proposes a TDOA-based localization algorithm for an underwater acoustic sensor network using the maximum-likelihood (ML) ratio criterion. To relax the complexity of the proposed localization complexity, we construct an auxiliary function, and use the majorization-minimization (MM) algorithm to solve it. The proposed localization algorithm proposed in this paper is called a T-MM algorithm. T-MM is applying the MM algorithm to the TDOA acoustic-localization technique. As the MM algorithm iterations are sensitive to the initial points, a gradient-based initial point algorithm is used to set the initial points of the T-MM scheme. The proposed T-MM localization scheme is evaluated based on squared position error bound (SPEB), and through calculation, we get the SPEB expression by the equivalent Fisher information matrix (EFIM). The simulation results show how the proposed T-MM algorithm has better performance and outperforms the state-of-the-art localization algorithms in terms of accuracy and computation complexity even under a high presence of underwater noise.

## 1. Introduction

Underwater acoustic sensor networks (UASNs) is a network composed of underwater sensor nodes randomly distributed in a certain observation area and uses underwater acoustic signals to communicate with each other. The research on underwater sensor networks has achieved rapid development in recent years. That is due to the wide range of UASNs applications from the petroleum industry to aquaculture, including instrument monitoring, pollution control, climate recording, natural interference prediction, search and investigation tasks, and marine biological research. Some applications such as marine resource exploration, military reconnaissance, underwater target monitoring and tracking, disaster prevention, and navigation [1,2] are required to locate the underwater target, as the purpose of the sensor network is not limited to only collect the underwater related data. Especially in disaster prevention applications, UASNs can promptly find seismic activity from remote areas through acoustic source localization, and provide earthquake or tsunami warnings for seaside areas. In addition, in order to improve communication and network performance, location information is also very useful for improving technologies such as topology control, routing, and packet collision avoidance [3]. Therefore, the problem of underwater acoustic source localization has become the key technology of UASNs and the most popular research hotspot in recent years.

However, due to the complexity of the marine environment, underwater positioning faces enormous difficulties and challenges compared to terrestrial wireless positioning [4,5]. The major difference between UASNs and terrestrial wireless positioning is the propagation signal types; the transmission signal used in UASNs is acoustic while the radio frequency is the transmission signal for the terrestrial wireless communication. The electromagnetic waves can only propagate through seawater over long distances at very low frequencies (30~300 Hz), and such low-frequency of radio signals require longer antennas and high transmission power. In the acoustic underwater data transmission, the acoustic signal attenuation is small hence it can support long-transmission distance, but the low speed of sound in ocean water (approximately 1500 m/s) yielding large propagation delay. Also, the acoustic channel bandwidth is limited, and factors such as multipath, channel fading, noise interference, and Doppler frequency shift will cause high channel error rate or even interruption. Hydroacoustic sensor nodes are affected by environmental factors such as ocean currents, temperature, salinity, and uncontrollable motion, and sensor nodes movement caused by water flow is inevitable. Due to all of the above challenges, underwater localization is an unease task, and a lot of researches is still needed at this point to improve the underwater localization techniques.

There are many types of research work on the acoustic-based localization, but most of these researches are applied on terrestrial applications and most of these algorithms are not suitable for underwater acoustic source localization. Nonetheless, there are some methods suitable for underwater (see [6,7]). In these positioning methods, the position of the underwater sensor node needs to be known in advance. These position-knowing nodes are called anchors in some works of literature [8,9], and the target nodes are called agents.

In [10], the author proposes an auxiliary positioning scheme based on the angle of arrival (AOA) for underwater Ad-Hoc sensor networks in 2-D and 3-D. This AOA acoustic localization technique classified the underwater nodes for sensor nodes and anchor nodes. Only one of the sensor nodes can communicate with the first anchor node in this scheme and estimate the distances between other sensor nodes and anchor nodes. The estimated distance is calculated with AOA assistance. In [11] authors have proposed a two-step acoustic-based localization technique based on improved received signal strength (RSS). In this scheme, the anchor nodes are placed on the water surface instead of underwater. The base station first uses a regression model to calculate the distance through the RSS information collected by the anchor node, and then roughly estimates the location information. Finally, the position is adjusted by minimizing the relevant mean square error. Underwater acoustic node location-based on the time of arrival (TOA) ranging strategy is proposed in [4]. In this scheme, the authors consider the problem of the underwater sensor motion due to the water flow. In this research, the grey wolf optimization algorithm has been proposed to estimate the optimal location of the underwater node’s and underwater displacement. In addition to the main acoustic-based localization schemes mentioned above, there are many other methods such as range-free [6] localization scheme.

The current acoustic-based localization methods mentioned above have many drawbacks, for example, the AOA-based algorithm may be affected by a severe Doppler shift in the underwater acoustic channel. As the underwater power loss is dependent on the communication distance and carrier frequency, the RSS-acoustic localization method results in ambiguous. The underwater acoustic-based TOA scheme requires very precise time synchronization, which is uneasy to be satisfied in the underwater communication. Although the localization method based on sound line correction [12] is more realistic, unfortunately, it did not further optimize the position to reduce the error. Due to the shortcoming in the current underwater localization, we applied the majorization-minimization (MM) algorithm based on time difference of arrival (TDOA) localization method to further optimization for the acoustic source localization. Using the TDOA acoustic-localization, the proposed algorithm in this paper is capable to estimate the location of the sensors only by using the measured time difference between the signals arrived at different sensors hence it is not affected by power loss and Doppler shift, and it also does not need accurate time synchronization. Due to all these features, the TDOA is more fitted for underwater acoustic-based localization. To improve the estimation accuracy of the proposed scheme, the MM algorithm is applied to the TDOA estimation. The MM-algorithm combination with the TDOA not only improve the estimation accuracy, but it also provides more accurate position information. Due to the high sensitivity of the MM-algorithm to the initial points, the initial point is set based on an initial point gradient algorithm. The proposed combined TDOA-MM (T-MM) algorithm with an assistant of an initial point gradient algorithm avoid the current drawbacks of the conventional acoustic-based localization.

In this paper, we assume that the sensor nodes know their axes coordinates in advance via global positioning system (GPS) and buoy, or by using the same method proposed in the literature [3]. We also derive and study the corresponding squared position error bound (SPEB) derived by Cramer-Rao lower bound (CRLB). Numerical simulations have also been conducted for evaluating the localization performance of our proposed algorithm. The main contributions of this paper can be summarized as follows.

(1) We investigated the unconstrained optimization problem based on least squares (LS) and maximum likelihood (ML) localization and applied it to the TDOA acoustic-based localization in the underwater applications. The localization accuracy of our proposed scheme is characterized and evaluated by using the SPEB.
(2) The proposed T-MM algorithm is using a multistep localization scheme by dividing the underwater location operation into three steps. First, estimate the distance between the acoustic source and sensor nodes by using the TDOA estimation algorithm. Second, the initial point is adjusted for the MM-algorithm by using an initial point gradient algorithm to improve the MM-algorithm operation. In the third operational step, the obtained initial point is used via the MM-algorithm to improve the localization accuracy.
(3) In this paper, we drove a mathematical framework for the proposed T-MM acoustic-based localization algorithm.
(4) We compare the performance of the proposed T-MM algorithm in terms of estimation accuracy with some of the state of the art acoustic-based localization techniques used currently in the underwater localization. Based on the simulation results, the performance of the proposed T-MM algorithm is more superior even under high underwater noise and its performance is evaluated by using the SPEB metric.

This paper is organized as follows: We first review the related work in Section 2. Section 3 demonstrates the system model and showing the communication Model. Section 4 mainly explains the optimization problem of localization and the proposed MM algorithm to solve it. In Section 5, the proposed T-MM is proposed and then derived the SPEB as a metric to evaluate its positioning performance. Finally, simulation results and performance analysis are given in Section 6, and conclusions are drawn in Section 7.

Notation: The following notations are used in this paper. ${\mathbb{R}}^{n}$ indicates an n-dimensional real space; upper bold-face letters stand for matrices and lower bold-face letters stand for vectors; ‖*‖ denotes the Euclidean distance norm of vectors *; ∇f indicates the gradient of function f; E[•] denotes the expectation operator; superscript T denotes the transpose of a matrix (vector); tr(*) denotes the trace of a square matrix *; ${\left[*\right]}_{n\times n}$ is the upper left n×n submatrix of matrix *; ${*}^{-1}$ denotes the inverse of the matrix *; rank(*) indicates rank of matrix *.

## 2. Related Works

In this section, we briefly overview the current localization techniques using the TDOA applied for the underwater wireless sensor networks. In addition to the estimation techniques used for optimizing the acoustic source position and improve the acoustic-based localization in the underwater environments.

TDOA locates the acoustic source by detecting the time difference between signals reaches two sensors. The TDOA reduces the time synchronization requirement between the acoustic source and the receiver sensor, but unfortunately, current TDOA is still required synchronization between the receiver sensors. In [13] the author uses AUV as a mobile anchor. By receiving the broadcast signal from the AUV, the sensor node can measure the arrival time of the received data packet and obtain a series of nonlinear equations. Then the author uses a two-phase algorithm for localization, in the first phase, the author transforms the nonlinear equations into linear equations and uses the least squares method to obtain coarse time synchronization results. The second phase uses another least square estimator technique to refine the results obtained in the first phase. The time synchronization algorithm proposed in [14] has considered the mobility of sensor nodes in the underwater wireless sensor networks (UWSN). To consider the node mobility the authors in [14] designed two mobile reference nodes to eliminate its adverse effects. In addition, the author designed the motion trajectories of two reference nodes in order to ensure the synchronization accuracy.

In [15] authors have been proposed an acoustic-based localization method for positioning algorithm in the shallow water environment. In this literature, the authors propose a closed-loop tracking algorithm based on Kalman filtering to track the peak of the time correlation of the received signals using two hydrophones. In this scheme, the TDOA is calculated for the acoustic signal through the analysis of the correlation between signals to estimate the sensor position. However, the author only used two hydrophones for receiving signals for TDOA measurements and reduce the positioning system accuracy.

Underwater positioning methods based on the frequency difference of arrival (FDOA) and TDOA have been proposed in [16] to address the acoustic-based localization in underwater environments. It considers the features of the acoustic signal propagation under undetermined acoustic speed propagation in the water. In this way, the influence of the Doppler effect is reduced with significant improvement for localization accuracy. However, the first-order Taylor approximation is applied during TDOA measurement plus linearized TDOA and FDOA greatly increases the complexity. High complexity acoustic-based localization system is unfortunately unacceptable as the underwater sensor nodes is a battery-based communication system and recharging capability is hard due to ocean environments [17,18,19].

In [20] authors uses both acoustic and optical signals to locate and track a target ship. The acoustic and optical link are used for the non-line-of-sight and the line-of-sight localization respectively. This hybrid system enables precise positioning tracking and high-speed underwater data transmission, but increases the cost of implementation because the target and AUV must be equipped with a hybrid acoustic-optical transceiver and high cost localization system restricts large-scale applications.

In [21], two localization algorithms called distance-based and angle-based algorithms for underwater acoustic localization have been proposed. In this scheme, four anchor nodes are placed at the four vertices of the square area, and the positions of the randomly placed mobile nodes are estimated. However, many iterations are required for the system implementation, and severe underwater multipath effects can affect the angle-based algorithm.

An algorithm named second-order time difference of arrival (STDOA) has been applied in finding the black box that was proposed in [22]. STDOA is defined as the difference of TDOA and it eliminates the unknown signal period. Nonetheless, this algorithm has limitations as it proposed to estimate only the location of the underwater black box. In other words, it is only suitable for estimate the location of sources transmits acoustic signals at a certain period.

There are conventional techniques in estimation theory that can be applied to estimate a target localization. These techniques such as LS estimator, convex optimization, and ML estimator are combined with the TDOA localization scheme. The combined TDOA localization technique with the optimization algorithms is a semi-definite programming (SDP) in [23,24], TDOA-LS based in [25,26], and TDOA-ML acoustic-based localization in [27]. The SDP-based method relaxes the original non-convex problem onto a convex set, thus effectively solving the new optimization problem [28]. However, the SDP model assumes that the input data are noise-free, this is not a realistic assumption because sensor measurements are often noisy. Moreover, for a good estimation accuracy, the relaxation needs to be very tight, which is rather challenging, not to mention the high computational complexity for solving the final problem. LS method is a very useful method used to solve TDOA positioning problem and in [25], the squared-range based LS formulation is exploited. Then the problem is converted into a known class of optimization problems, namely generalized trust region sub-problem (GTRS), using the concepts in robust statistics. Two algorithms are proposed to solve this GTRS problem, called squared range iterative reweighted least-squares (SR-IRLS) and squared range gradient descent (SR-GD) algorithm. Unfortunately, the two algorithms have computation complexity and high computation complexity is unacceptable in the underwater applications due to energy restrictions. In another related literature [26], the author first converts the obtained squared range least square (SR-LS) optimization problem into a constrained optimization problem and then try to solve the optimization problem by discarding the quadratic constraints. Several algorithms based ML criteria were also reported in [27], unfortunately, these localization algorithms are a tradeoff between the complexity and accuracy.

Therefore, this paper introduces the MM optimization algorithm in combination with TDOA localization for underwater acoustic localization. The proposed scheme improves the accuracy of the underwater positioning, in addition to, relax the system computation complexity compared with the state of the art acoustic-based localization techniques used in underwater applications.

## 3. Preliminaries

In this section, the detailed scenario of the underwater sensors network and the communication model of the UASNs will be presented. First, the system model of the UASNs is discussed and presented in detail. Then, the detailed underwater acoustic channel parameters are represented. Based on that, the TDOA fitted for acoustic-based localization in the multipath underwater acoustic channel is shown.

### 3.1. System Model of the Underwater Acoustic Sensors Networks

**Figure 1:**
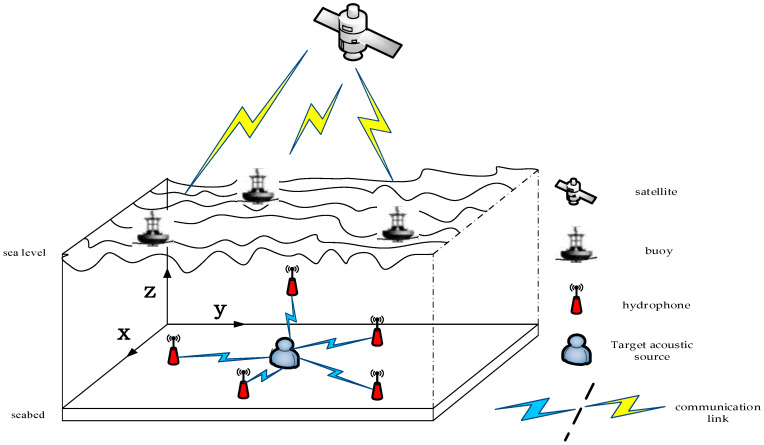
3-dimension model for the underwater acoustic sensor networks (UASNs). Each hydrophone receives the signal from the targeted acoustic source.

The proposed acoustic-based localization can be applied in the UASNs communication scenario shown in Figure 1. In the UASNs as usual, a pre-known location for several sensors (anchors) is needed. The anchors may be placed at a fixed location and their coordinates may have been pre-configured, or they may be floated and included special hardware to learn their locations from a location server, such as GPS. Anchors can also obtain their own coordinate information using a self-localization algorithm as in [3]. In the 3-dimension model of UASNs shown in Figure 1, the surface buoy can estimate its location by using a satellite system and provide its positioning information to the anchors. The localization operations of the proposed algorithm will be used to estimate and measure the distance between the anchors and the target (agent). The target is randomly placed in the measuring area and we assume it is fixed for the localization estimation time. The underwater hydrophones have a fixed location known by the localization service. Unlike the black box localization proposed in [22] the target in our paper has a random transmission period and the localization system does not have a pre-information about the transmitted signal.

### 3.2. Communication Model of the UASNs

The underwater acoustic channel characteristics have been addressed in many kinds of literature. In this sub-section, the feature characteristics of the underwater acoustic channel are briefly presented. The underwater acoustic channel loss depends on the distance between the transmitter and receiver and based on the signal carrier frequency. The signal carrier frequency of the signal determines the absorption loss due to the conversion of acoustic energy into heat through the wave propagation. The absorption loss increases with the increase of frequency and distance and ultimately puts a limit on the available bandwidth under the practical constraints of limited transmission power.

The classic signal structure is mainly a positioning signal with sine and cosine as carriers, but it is not suitable for underwater acoustic channels. Therefore, in this paper, we use linear frequency modulation (LFM) signal as a positioning signal, that is, the signal from the acoustic source is assumed to be LFM and can be written as:

(1) $$x_{s}\left(t\right)=A_{0}rect\left(\frac{t}{T}\right)e^{j2\pi \left(f_{0}t+\frac{1}{2}kt^{2}\right)},$$

where $A_{0}$ is the amplitude of signal and $rect\left(\frac{t}{T}\right)$ is the rectangular window function. The phase of the signal at time t is $2\pi \left(f_{0}t+\frac{1}{2}kt^{2}\right)$. T and $f_{0}$ are the pulse width and initialization frequency of the signal, respectively. $k=\frac{B}{T}$ is the frequency modulation rate of the signal, it represents the frequency change value per unit time and B is singal’s bandwidth. By differentiating the phase in time, the instantaneous frequency of the signal can be obtained as $f=f_{0}+kt,0\ll t\ll T.$Therefore, the received signal by the sensor can be written as:

(2) $$x_{i}\left(t\right)=A_{i}rect\left(\frac{t}{T}\right)e^{j2\pi \left(f_{0}t+\frac{1}{2}kt^{2}\right)+j\theta_{i}}+\omega_{i}\left(t\right),$$

where i=1, 2, …,m represents the number of sensors. $A_{i}$ is the amplitude of the received signal in i-th sensor which can be obtained by Equation (3) in [20], $\theta_{i}$ is the phase shift caused by propagation delay. $\omega_{i}\left(t\right)$ is an additive zero-mean Gaussian random noise. In this paper, we estimate the location of a fixed underwater target without considering the relative motion of the sensor and the target, so we do not consider the Doppler effect.

Attenuation of the underwater acoustic channel for a transmitted signal with a certain frequency over a transmission distance is given as [29]:

(3) $$A\left(l,f\right)=A_{norm}l^{k}a{\left(f\right)}^{l},$$

where $A_{norm}$ is a normalization constant, a(f) is the absorption coefficient, k is the spreading factor that is used to describes the geometry of propagation, and its usually used values is k=2 for spherical spreading, k=1 for cylindrical spreading, and k=1.5 for practical spreading. Underwater attenuation expressed in dB is given by

(4) $$10\;log\;A\left(l,f\right)=k\cdot 10\log l+l\cdot 10\log a\left(f\right)+10\log A_{norm},$$

The term k⋅10logl represents the spreading loss, and the term l⋅10loga(f) represents the absorption loss. The absorption coefficient a(f) is expressed empirically for frequency values above a few hundred Hz as follows:

(5) $$10\;log\;a\left(f\right)=0.003+\frac{0.11f^{2}}{1+f^{2}}+\frac{44f^{2}}{4100+f^{2}}+2.75\cdot 10^{-4}f^{2},$$

where 10loga(f) is given in dB/km. Equation (5) can be simplified at low carrier frequency as:

(6) $$10\;log\;a\left(f\right)=0.002+0.11\frac{f^{2}}{1+f^{2}}+0.011f^{2},$$

**Figure 2:**
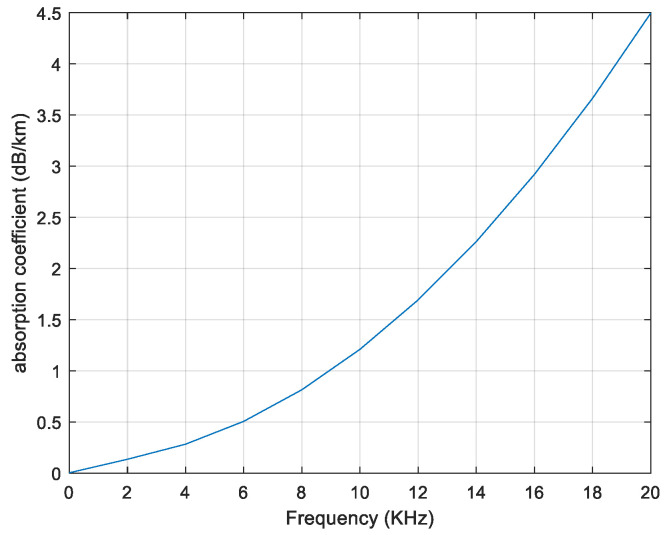
Absorption coefficient 10loga(f).

The underwater acoustic channel is a frequency-dependent channel, the relationship between the underwater absorption coefficient and the signal carrier frequency is shown in Figure 2.

As shown in Figure 2, the absorption coefficient increases rapidly with frequency increasing. Therefore, due to the limitation of the bandwidth of the underwater acoustic channel and the time-varying nature of the channel, many positioning algorithms are inaccurate or even invalid. For example, the Doppler shift will affect the AOA algorithm, and the underwater power loss (depending on distance and frequency) makes the RSS-based method results ambiguous. Intuitively, TOA-based and TDOA-based estimation are both feasible for underwater acoustic source localization. But TOA-based estimation requires very precise time synchronization, which is difficult to achieve accurate underwater localization. Consequently, it is reasonable to choose TDOA, which is easier to implement and used in underwater acoustic-based localization algorithms.

## 4. Proposed Localization Algorithm Based on Majorization-Minimization Optimization

In this section, the details of the proposed acoustic-based localization system are explained. First, the problem is formulated and discussed in detail. Then, the detailed TDOA acoustic-based localization algorithm for the underwater wireless sensor network is explained and discussed. Finally, the MM optimization technique is formulated for the TDOA acoustic-based localization.

### 4.1. Problem Formulation

Assume there is a UASN that has several hydrophones as depicted in Figure 1, as explained in the system model of the underwater acoustic sensors networks sub-section, we assume the anchors are fully known their location information. The coordinate vector of the i-th underwater hydrophone can be denoted as $s_{i}\in {\mathbb{R}}^{n}$ (in practical applications n=2 or n=3). The $x\in {\mathbb{R}}^{n}$ represents the coordinates vector for the source target. So, we can get the following equation:

(7) $$d_{i}=\|x-s_{i}\|+z_{i},\;\;i=1,2,3....m,$$

where $d_{i}$ denotes the distance between the acoustic source and the hydrophone node, and $z={\left(z_{1},z_{2},\ldots z_{m}\right)}^{T}$ denotes measurement noise error vector. We assume that measurement error $z_{i}$ be independent and identically distributed Gaussian random variables, $z_{i}∼N\left(0,\delta^{2}\right)$. Such observations can be obtained from the TOA or TDOA measurement. Thus, the underwater acoustic source localization problem can be described as: given the observed range measurement $d_{i}>0$, find a “good” approximation of the source x [30]. By applying the least squares criterion for the model in Equation (7), we can get the optimization problem as:

(8) $$\underset{x\in {\mathbb{R}}^{n}}{min}\left\{f\left(x\right)\equiv \overset{m}{\underset{i=1}{\sum }}{\left(d_{i}-\|x-s_{i}\|\right)}^{2}\right\},$$

In fact, the above optimization problem is the maximum likelihood ratio estimation [31]. The noise vector z follows the Gaussian distribution with a covariance matrix proportional to the identity matrix and the source x in the ML algorithm is estimated for solving the problem in Equation (8). The above optimization problem is a nonconvex optimization problem due to $-2d_{i}\|x-s_{i}\|$ term. This optimization equation can be solved by using many optimization algorithms. Throughout this paper, we denote the set of sensors by $\Psi =\left\{s_{1},s_{2},\ldots ,s_{m}\right\}$. In the following content, will be explained in detail to show how the TDOA algorithm can be applied for acoustic-based localization.

### 4.2. TDOA-Based Measurement Using Straight Line Propagation Model

The speed of sound in water is not constant, it is affected by temperature, salinity, and depth. Therefore, the propagation path is not a straight line but a curve. However, these factors only have a small impact on sound speed over a short-range. Thus, we assume that sound speed is approximately constant, and we use a straight-line propagation model in this paper.

**Figure 3:**
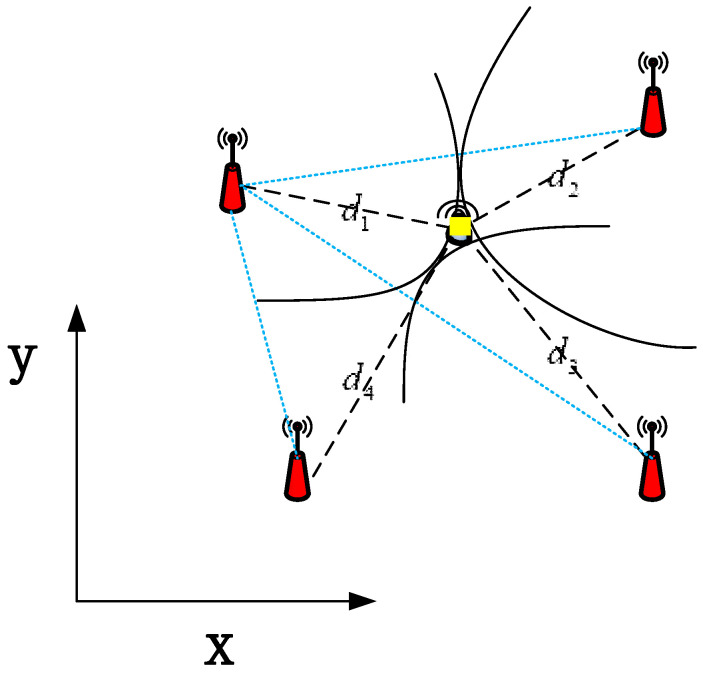
Time difference of arrival (TDOA) in 2-dimension underwater plane (the yellow area is the location of the acoustic source).

TDOA is a localization method based on the arrival time difference. By comparing the absolute time difference of the acoustic source to each anchor, a hyperbola with the anchor focus and the distance difference as the long axis can be made (see Figure 3). The intersection point of the hyperbola is the location of the acoustic source. Next, we will talk about the TDOA-based algorithm in detail. Let $t_{i}$ be the time of transit from the source to i-th hydrophone, and let $v_{water}$ be the underwater speed of sound (1500 m per second in water). Then, $d_{i}=v_{water}t_{i}$, is the distance between the acoustic source and the i-th hydrophone. Therefore, $\tau_{i}=t_{i}-t_{1}+n_{i}$, is the difference in transit time between hydrophone i-th and first hydrophone and can be obtained by using some algorithms, where $n_{i}$ is the zero-mean Gaussian TDOA noise.

Note that,

(9) $$v_{water}\tau_{i}=v_{water}t_{i}-v_{water}t_{1}=d_{i}-d_{1},$$

therefore,

(10) $$d_{i}^{2}={\left(v_{water}\tau_{i}+d_{1}\right)}^{2}=v_{water}^{2}\tau_{i}^{2}+2d_{1}v_{water}\tau_{i}+d_{1}^{2},$$

we move everything over to get,

(11) $$v_{water}\tau_{i}+2d_{1}+\frac{d_{1}^{2}-d_{i}^{2}}{v_{water}\tau_{i}}=0,$$

for i=2, 3…m. Then we can get,

(12) $$v_{water}\tau_{i}-v_{water}\tau_{2}+\frac{d_{1}^{2}-d_{i}^{2}}{v_{water}\tau_{i}}-\frac{d_{1}^{2}-d_{2}^{2}}{v_{water}\tau_{2}}=0,.\;$$

where i=3, 4,…m.

For simplicity, we assume n=2, which means the positioning is estimated in two-dimensional plane. By substitute $d_{i}=\sqrt{{\left(x_{i}-x\right)}^{2}+{\left(y_{i}-y\right)}^{2}}$ into the above equations $d_{1}^{2}-d_{i}^{2}$ we can obtain:

(13) $$d_{1}^{2}-d_{i}^{2}=x_{1}^{2}+y_{1}^{2}-x_{i}^{2}-y_{i}^{2}-2xx_{1}-2yy_{1}+2xx_{i}+2yy_{i},$$

for i=3, 4,…m. By solving the above result into Equation (12) we can obtain:

(14) $$D_{i}+A_{i}x+B_{i}y=0,$$

where,

(15) $$A_{i}=\frac{1}{v_{water}\tau_{i}}\left(-2x_{1}+2x_{i}\right)-\frac{1}{v_{water}\tau_{2}}\left(-2x_{1}+2x_{2}\right),$$

(16) $$B_{i}=\frac{1}{v_{water}\tau_{i}}\left(-2y_{1}+2y_{i}\right)-\frac{1}{v_{water}\tau_{2}}\left(-2y_{1}+2y_{2}\right),$$

(17) $$D_{i}=v_{water}\tau_{i}-v_{water}\tau_{2}+\frac{1}{v_{water}\tau_{i}}\left(x_{1}^{2}+y_{1}^{2}-x_{i}^{2}-y_{i}^{2}\right)-\frac{1}{v_{water}\tau_{2}}\left(x_{1}^{2}+y_{1}^{2}-x_{2}^{2}-y_{2}^{2}\right),$$

The above formula can be written in matrix form as follows:

(18) $$\left[\begin{array}{c} A_{3}\;B_{3}\\ \vdots \;\vdots \\ A_{i}\;B_{i} \end{array}\right]\cdot \left[\begin{array}{c} x\\ y \end{array}\right]=\left[\begin{array}{c} -D_{3}\\ \;\vdots \\ -D_{i} \end{array}\right],$$

and i=3, 4,…m. The Moore-Penrose pseudoinverse can be applied at both sides of the matrix in Equation (18), to solve the equation in the x−y plane and the distance $d_{i}$ will be calculated accordingly. Due to the underwater environment complexity, TDOA acoustic-based localization has low localization accuracy and it has a measurement error. So, optimization techniques are required to improve the accuracy of the TDOA acoustic-based localization. TDOA localization algorithm is shown in detail in Algorithm 1.
**Algorithm 1** TDOA measurements in 2-D underwater planeInput: Coordinate $\left(x_{i},y_{i}\right)$ of hydrophone. Output: (x,y). Steps: 1: Calculation time difference by $\tau_{i}=t_{i}-t_{1}$. 2: Substitute $\tau_{i}$ into Equations (15)–(17) to get $A_{i},B_{i},$
$D_{i}$. 3: Substitute $A_{i},B_{i},D_{i}$ into (14) to get (x,y). 4: End 

### 4.3. Majorization-Minimization Algorithm Based TDOA

In this paper, we apply MM algorithm to solve the optimization problem in Equation (8) and improve the accuracy of the TDOA acoustic-localization scheme. The combined TDOA and MM algorithm, called T-MM in this paper. According to research work in [32], we need to construct a continuous surrogate function satisfied with the following conditions; (1) $M\left(x,x\right)=f\left(x\right)\;\forall x\in {\mathbb{R}}^{n},x\notin \Psi$; and (2) $M\left(x,y\right)\geq f\left(x\right)\;\forall x,y\in {\mathbb{R}}^{n},y\notin \Psi$. Hence, we construct an auxiliary function as follows:

(19) $$M\left(x,y\right)\equiv \overset{m}{\underset{i=1}{\sum }}\|x-s_{i}-d_{i}{\frac{y-s_{i}}{\left\|y-s_{i}\right\|}\|}^{2}\;\forall x,y\in {\mathbb{R}}^{n}\;\mathrm{and}\;y\notin \Psi ,$$

and evaluate this auxiliary based on the two conditions (1) and (2) mentioned above. The first condition can be proved using a simple mathematical algebra as follows, for every x∉Ψ, we have:

(20) $$\;M\left(x,x\right)=\overset{m}{\underset{i=1}{\sum }}\|x-s_{i}-d_{i}{\frac{x-s_{i}}{\left\|x-s_{i}\right\|}\|}^{2}={\left\|\frac{\left(x-s_{i}\right)\left(\|x-s_{i}\|-d_{i}\right)}{\left\|x-s_{i}\right\|}\right\|}^{2}=\overset{m}{\underset{i=1}{\sum }}{\left(\|x-s_{i}\|-d_{i}\right)}^{2}=f\left(x\right),$$

In order to prove the second condition, let us subtract the following two functions as follows:

(21) $$\begin{array}{ll} M\left(x,y\right)=\overset{m}{\underset{i=1}{\sum }}\|x & -s_{i}-d_{i}{\frac{y-s_{i}}{\left\|y-s_{i}\right\|}\|}^{2}=\overset{m}{\underset{i=1}{\sum }}\left\{{\left(x-s_{i}-d_{i}\frac{y-s_{i}}{\left\|y-s_{i}\right\|}\right)}^{T}\left(x-s_{i}-d_{i}\frac{y-s_{i}}{\left\|y-s_{i}\right\|}\right)\right\}\\ & =\overset{m}{\underset{i=1}{\sum }}\left\{\|x-s_{i}\|{}^{2}-2d_{i}{\left(x-s_{i}\right)}^{T}\frac{y-s_{i}}{\left\|y-s_{i}\right\|}+d_{i}^{2}\right\}, \end{array}$$

(22) $$f\left(x\right)=\overset{m}{\underset{i=1}{\sum }}{\left(d_{i}-\|x-s_{i}\|\right)}^{2}=\overset{m}{\underset{i=1}{\sum }}\left(d_{i}^{2}+\|x-s_{i}\|{}^{2}-2d_{i}\|x-s_{i}\|\right),$$

(23) $$M\left(x,y\right)-f\left(x\right)=\overset{m}{\underset{i=1}{\sum }}2d_{i}\left(\|x-s_{i}\|-{\left(x-s_{i}\right)}^{T}\frac{y-s_{i}}{\left\|y-s_{i}\right\|}\right)\geq 0,$$

where the last inequality follows from Cauchy-Schwartz inequality [32]. Consequently, $x_{k}$ can be updated as:

(24) $$\underset{x}{x_{k}=argmin\;}M\left(x,x_{k-1}\right),$$

If the gradient of $M\left(x,x_{k-1}\right)$ function is equal to zero, we can get:

(25) $$\nabla M\left(x,x_{k-1}\right)=\overset{m}{\underset{i=1}{\sum }}2\left(x-s_{i}-d_{i}\frac{x_{k-1}-s_{i}}{\left\|x_{k-1}-s_{i}\right\|}\right)=0,$$

and,

(26) $$x_{k}=\frac{1}{m}\overset{m}{\underset{i=1}{\sum }}\left(s_{i}+d_{i}\frac{x_{k-1}-s_{i}}{\left\|x_{k-1}-s_{i}\right\|}\right).$$

Note that two formulas are used in the above proof and

(27) $$\frac{\partial \left(x^{T}Ax\right)}{\partial x}=\left(A+A^{T}\right)x,\|x\|=\sqrt{x^{T}x}.$$

Based on the expression of the M(x,y) function, if y∈Ψ, then the function is meaningless. By choosing a suitable initial point $x_{0}$, we can ensure that the generated solution during the iteration process will not appear in the Ψ sensor set. The relevant proof derivation results were seen in the literature [27], and the detailed initial point algorithm based initial gradient algorithm will be shown below.

## 5. Initial Point and Performance Metrics

Here, we first give the initial point algorithm that provided from [27] and applied it to the proposed T-MM algorithm. Then we prove the convergence of the $f\left(x_{k}\right)$. Finally, we derive the squared position error bound (SPEB) as the underwater localization performance metric.

### 5.1. Initial Point Design and T-MM Convergence

The descent directions provided by 2.5 Lemma in [27], can be used to compute the initial point $x_{0}$ satisfied,

(28) $$f\left(x_{0}\right)<\underset{i=1,\ldots ,m}{min}f\left(s_{i}\right).\;$$

The detailed initial point algorithm is shown in Algorithm 2.

In Algorithm 2, $g_{j}\left(x\right)=\sum_{i=1,i\neq j}^{m}{\left(\|x-s_{j}\|-d_{i}\right)}^{2}$, where j=1,…,m and e is the vectors of all ones (we also chosen any other nonzero vector).
**Algorithm 2** an initial point algorithmInput: t=1. Output: $x_{0}=s_{k}+tv_{0}$. Steps: 1: Set k to be an index for which $f\left(s_{k}\right)=\underset{i=1,\ldots ,m}{min}f\left(s_{i}\right)$. 2: Set    $v_{0}=\{\begin{array}{c} -\nabla g_{k}\left(s_{k}\right)\nabla g_{k}\left(s_{k}\right)\neq 0,\\ e\nabla g_{k}\left(s_{k}\right)=0, \end{array}$ 3: While $f\left(s_{k}+tv_{0}\right)\geq f\left(s_{k}\right)$ 4: Set   $t=\frac{t}{2}$. 5: End 

Now we can easily prove the convergence of the $f\left(x_{k}\right)$ function. Equation (24) can be driven as:

(29) $$M\left(x_{k},x_{k-1}\right)\leq M\left(x_{k-1},x_{k-1}\right).\;$$

and hence from first and second conditions, the $f\left(x_{k}\right)$ function should follow that:

(30) $$f\left(x_{k}\right)\leq M\left(x_{k},x_{k-1}\right)\leq M\left(x_{k-1},x_{k-1}\right)=f\left(x_{k-1}\right).\;$$

Obviously, $f\left(x_{k}\right)\geq 0$. So, the $f\left(x_{k}\right)$ function is bounded and non-increased. In other words, T-MM algorithm use Algorithm 2 as an initial point. The proposed acoustic-based localization algorithm joint T-MM with Algorithm 2 as described in detail in Algorithm 3.
**Algorithm 3** T-MM underwater acoustic-based location algorithm.Input: Set the initial point $x_{0}$ by Algorithm 2 and the parameter η. And calculate distance $d_{i}$ by Equation (7) mentioned in Section 4. Output: $x_{t}$ Steps: 1: Set the number of iterations $t_{max}$; 2: Set t=0; 3: if $t\leq t_{max}$; 4:  if $\|\nabla f\left(x_{t})\right\|\leq \eta$
5:   Go to step 9; 6:  else if 7:   update $x_{t}$ as (24); 8:   t=t+1; 9: end if

### 5.2. Squared Position Error Bound (SPEB)

In this paper, we use the SPEB as an indicator for the accuracy of positioning algorithms as in [8]. According to [33,34], the definition of the squared position error (SPE) for underwater target for underwater acoustic sensors network is:

(31) $$\rho \left(x\right)=E\left[\|\hat{x}-{x\|}^{2}\right],$$

where $\hat{x}$ is the estimated position. So we can get inequality according to [8] as:

(32) $$\rho \left(x\right)=E\left[\|\hat{x}-{x\|}^{2}\right]\geq \mathrm{tr}\left\{{\left[J^{-1}\left(x\right)\right]}_{n\times n}\right\},$$

where ${\left[J^{-1}\left(x\right)\right]}_{n\times n}$ is the equivalent Fisher information matrix (EFIM) for the estimated position x. ${\left[J^{-1}\left(x\right)\right]}_{n\times n}$ is the upper left n×n submatrix of $J^{-1}\left(x\right)$, where J(x) is Fisher information matrix of position x. In two-dimensional localization problem, since $x={\left(x,y\right)}^{T}$, we need 2×2 matrix ${\left[J^{-1}\left(x\right)\right]}_{2\times 2}$. Therefore, we define the right-hand side of (32) as a measure to characterize the limits of localization accuracy as follows:

(33) $$SPEB\left(x\right)=\mathrm{tr}\left\{{\left[J^{-1}\left(x\right)\right]}_{n\times n}\right\}$$

So, we need to figure out FIM J(x). About FIM, many articles and mathematical literature have introduced it and solved it in detail (see, e.g., [9,33,34,35]). Here we give the result of J(x) directly as follows:

(34) $$J\left(x\right)=\frac{1}{\delta^{2}}\overset{m}{\underset{i=1}{\sum }}y_{i}y_{i}^{T}$$

where $y_{i}=\frac{\left(x-s_{i}\right)}{x-s_{i}}$. From these literatures, we can know that the inverse matrix exists only if and only if the rank of Fisher information matrix equal to the dimension of sensor coordinate vector, its mean rank(J(x))=n and m≥n.

So the SPEB can be written as:

(35) $$SPEB\left(x\right)=\mathrm{tr}\left\{{\left[{\left(\frac{1}{\delta^{2}}\overset{m}{\underset{i=1}{\sum }}y_{i}y_{i}^{T}\right)}^{-1}\right]}_{n\times n}\right\},$$

and SPEB gives the best expectations of unbiased estimation, which provides a scale for the performance of our research algorithms.

## 6. Numerical Simulation and Discussion

In this section, the performance of the proposed acoustic-based localization scheme is evaluated by a statistical underwater acoustic channel model as well as the bellhop underwater channel model. Under the two-channel models, the performance of the T-MM acoustic-based localization based on the initial point explained in detail in Algorithm 2, is evaluated and tested. In this section, the proposed scheme is compared with the existing localization algorithms used in the underwater through Monte Carlo simulations. In this section, we will compare the proposed algorithm with SR-LS [26], unconstrained squared-range-based LS estimate (USR-LS) [26], substantively weighted least squares (SWLS) [27], and semidefinite relaxation (SDR) [24,26]. The mean square error (MSE) is used in this section to evaluate the performance of the proposed algorithm as $MSE\left(x\right)=\sum_{i=1}^{N}\frac{\|\hat{x}-{x\|}^{2}}{N}$, and the unit of MSE is square meters. x is the correct position of the target acoustic source, $\hat{x}\;$is the estimated position obtained using acoustic-based algorithms and N is the number of ensemble runs. In this simulation, we assume the number of sensors used to estimate the underwater acoustic source is 5, and their coordinates are set to be (2,−3) (1,−4) (7, 1) (−9, 5) (3, 2). Then the position of the target is set as x=(2,3). The hydrophone and target coordinates are also in meters.

### 6.1. Experiment 1: Statistical Underwater Acoustic Channel Model

**Figure 4:**
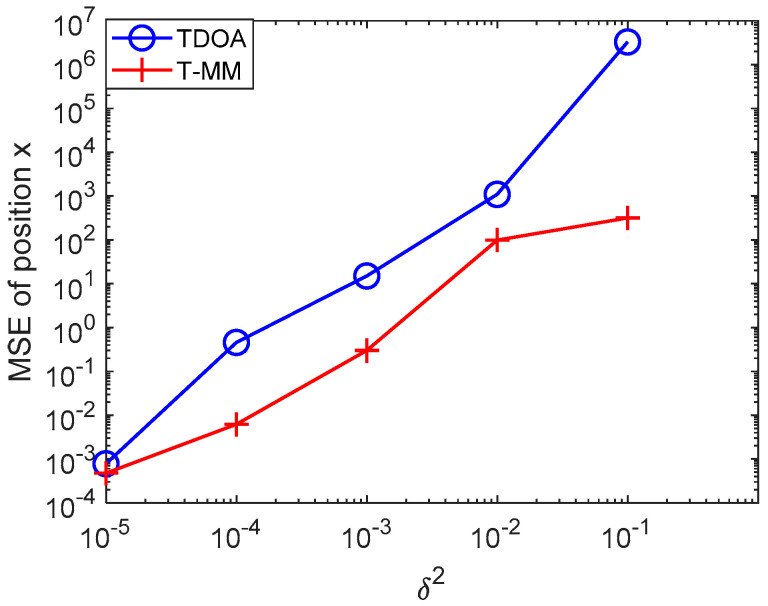
Mean square error (MSE) comparison between conventional TDOA and the proposed TDOA-majorization-minimization (T-MM) versus $\delta^{2}$.

In this simulation, we evaluate the proposed T-MM localization scheme over a statistical underwater acoustic channel model. We conducted 100 Monte Carlo trials and, in each realization, we extract another random set of noise value. We first verified the feasibility of the TDOA algorithm and the T-MM algorithm and then compared the conventional TDOA with the proposed T-MM algorithms. For the variance of TDOA measurement error, we take on: $10^{-5}$, $10^{-4}$, $10^{-3}$, $10^{-2}$, $10^{-1}$. As shown in Figure 4, the T-MM outperforms the conventional TDOA in terms of localization accuracy. Then we compared the proposed T-MM algorithm with some conventional localization algorithms and measured distances $d_{i}$ given by (7) with $z_{i}$. For variance $\delta^{2}$ of $z_{i}$, we take on five different values calculated from the SNR formula, $4.84\times 10^{-4}$, $4.84\times 10^{-3}$, $4.84\times 10^{-2}$, $4.84\times 10^{-1}$, 4.84 in our simulation and the SNR formula is defined as $10\log\left(\|{s\|}^{2}/\left(m\sigma^{2}\right)\right)$ [35]. For convenience, our simulation scenario considers n=2 dimensional. In general, each acoustic source can be equipped with a pressure sensor that can detect its depth information.

**Figure 5:**
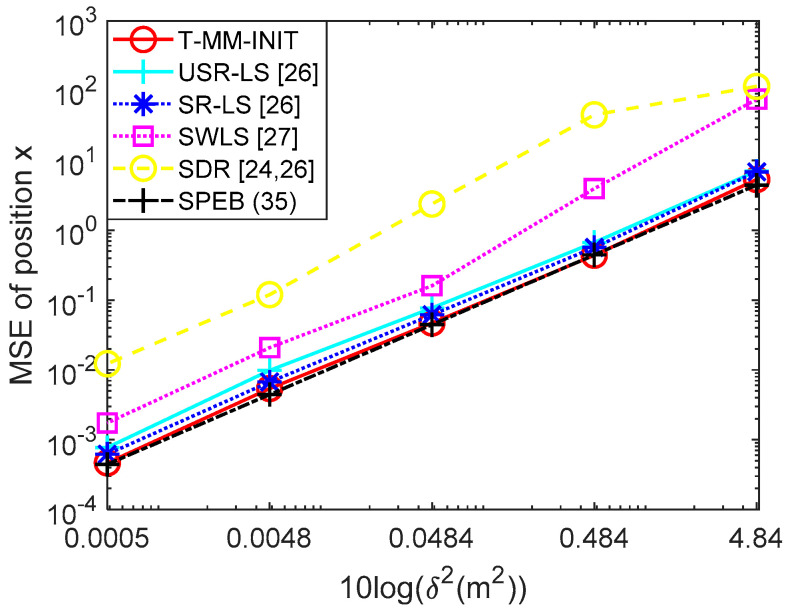
Conventional localization algorithm’s MSE comparison to the squared position error bound (SPEB) versus δ.

As shown in Figure 5, the performance of the proposed algorithm is evaluated under underwater noise. As shown in Figure 5, in case of increasing the underwater noise and compared with the SPEB. The SPEB is calculated based on Equation (35). As shown in Figure 5, the MSE of the proposed T-MM algorithm provided a localization accuracy nearly the same as the SPEB, even under high environmental noise. In other words, the T-MM is superior and robust to other algorithms. It is worth mentioning that the performance of algorithm SR-LS is very close to T-MM and as the noise level increases, the MSE of algorithm SWLS grows larger, which means that SWLS is less resistant to noise. The SDR performance is relative to other algorithms. See Table 1 for more details about the MSE’s value of all algorithms.

**Figure 6:**
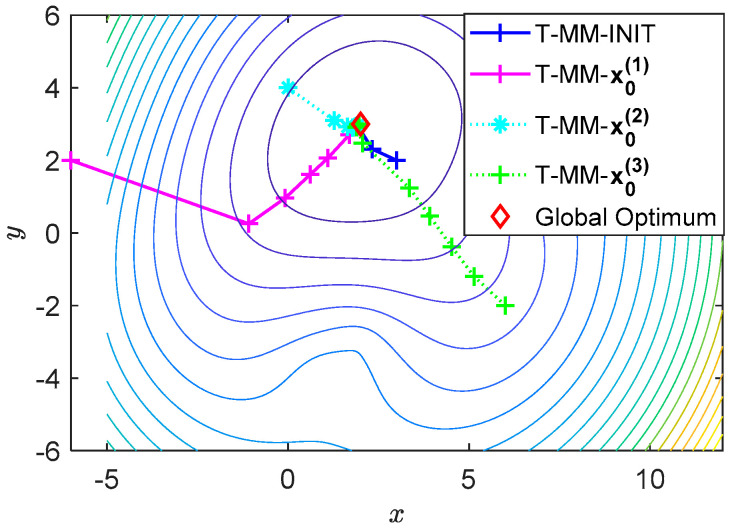
Start with four different initial points. True position of target is x=(2,3). Initial points calculated by the initial gradient algorithm is T-MM-INIT. The randomly selected initial points are $x_{0}^{\left(1\right)}=\left(-6,2\right)$, $x_{0}^{\left(2\right)}=\left(0,4\right)$, $x_{0}^{\left(3\right)}=\left(6,-2\right)$.

The performance of the prosed scheme is also evaluated under different numbers of iterations and convergence of the T-MM algorithm with different initial points, as depicted in Figure 6. For the T-MM algorithm, we selected four different initial points for iteration, one is calculated by the initial gradient algorithm, and the remaining three are randomly generated, they are coordinates (−6,2), (0,4), (6,−2) respectively. From the simulation results, we can see that when T-MM uses different initial points, the algorithm can quickly converge to the global optimal. All cases eventually converge to (1.9625, 2.9988), which is very close to the correct coordinates x=(2,3) of the acoustic source. This means the proposed T-MM algorithm is feasible for a suitable random initial point. But T-MM based on the initial gradient algorithm quickly converges to the global optimum, which takes about twenty-six steps. However, the risk of using a random initial point is the randomly selected point may be in the sensor set Ψ, which means that the algorithm will lose its effect.

**Figure 7:**
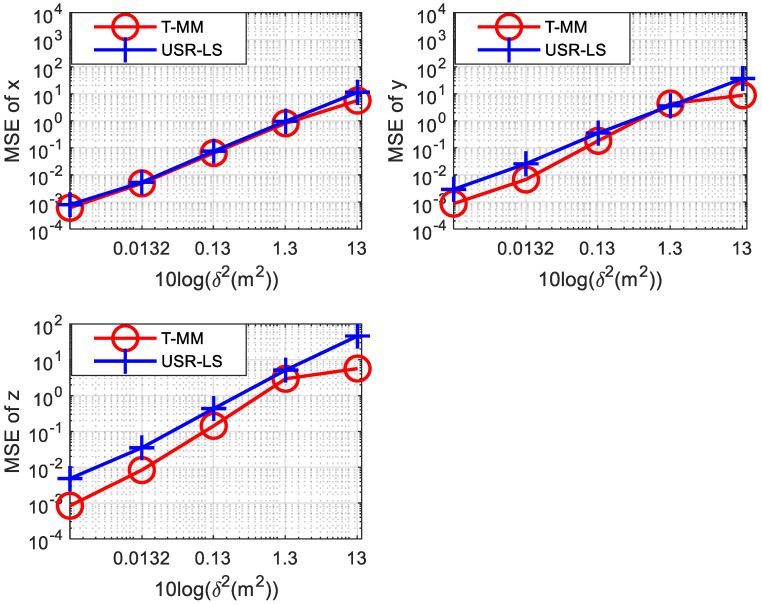
Comparison between the proposed T-MM scheme and conventional USR-LS localization scheme in terms of the MSE under different $\delta^{2}$ values in 3D scenario.

In a 3-dimensional scenario, the location information of the target sound source is a 3-dimensional vector. Similar to the 2-dimensional scenario, we also simulated underwater positioning in a 3-dimensional scenario scene. Since we need to calculate the three-dimensional information of the target sound source, we need more sensors. In the simulation we use seven sensors for 3D scene. Their coordinates are set to be (2,−3,1) (1,−4,3) (7, 1,2) (−9, 5,6) (3, 2,8)(10,−8,−10)(12,−6,−2). Then the position of the target is set to be x=(2,3,5). Other simulation settings are the same as in the 2-dimensional case mentioned above. As shown in Figure 7, we calculated the MSE of the acoustic source’s abscissa, ordinate, and vertical coordinate under the two algorithms, respectively. It can be seen from the three sub-graphs that the MSE of the T-MM algorithm is always smaller than the MSE of the USR-LS in the 3D scenario. In other words, the T-MM is superior to USR-LS.

Next, we have studied the influence of the number of underwater sensors on location accuracy. We analyzed the effect of positioning accuracy when the number of sensors is m = 4, 5, 6, 7 respectively.

**Figure 8:**
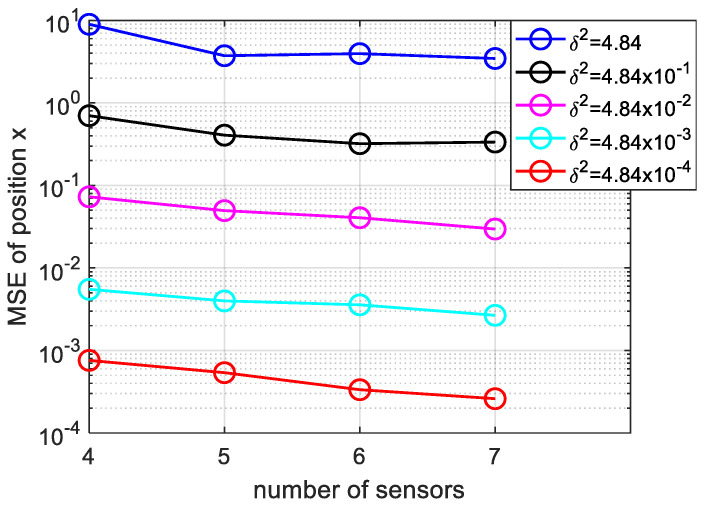
Variation of MSE of position x with more sensors and varying measurement noise, the sensors number is m = 4, 5, 6, 7.

Figure 8, shows that, with increasing the number of underwater sensors, the localization accuracy is improved. It is obvious, from the TDOA principles, the larger the number of sensors means more hyperbola and accurate positioning. From another point of view, the increase in sensors means that we obtain more information about the acoustic source, so the positioning accuracy improved and in practical UASNs, the number of sensor nodes can reach hundreds.

### 6.2. Experiment 2: Bellhop Underwater Channel Model

In order to verify the feasibility of the proposed T-MM acoustic-based localization over the underwater acoustic channel, in this subsection, we use the bellhop model which closer to the actual underwater environment for simulation. The bellhop model uses the Gaussian beam tracking method to calculate the sound field in a horizontal non-uniform environment, which can be used to simulate the underwater environment. The underwater environment parameter settings for the bellhop underwater acoustic channel used in this paper are listed in Table 2.

In the setting of environmental parameters above, the density of water column is 1021 kg/$m^{3}$, which is the density of sea water in summer. The density of bottom halfspace means that sound velocity of the sediment layer is 1810  kg/$m^{3}$.

**Figure 9:**
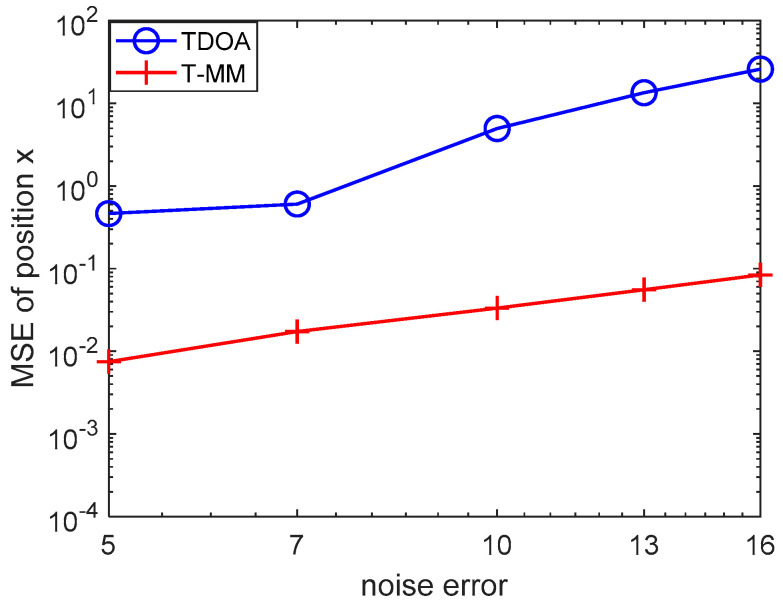
MSE comparison between conventional TDOA and the proposed T-MM versus measurement noise error over the bellhop underwater channel model.

**Figure 10:**
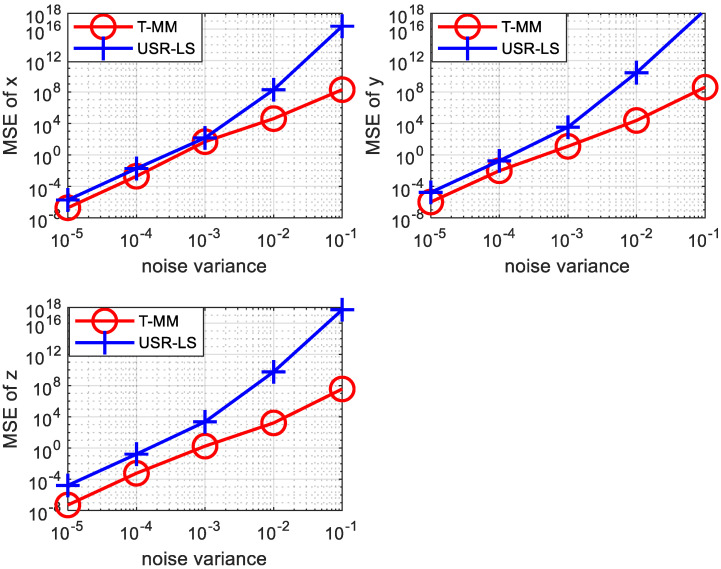
T-MM’s MSE comparison to the USR-LS’s MSE versus noise variance in 3D scenario over the bellhop underwater channel model.

**Figure 11:**
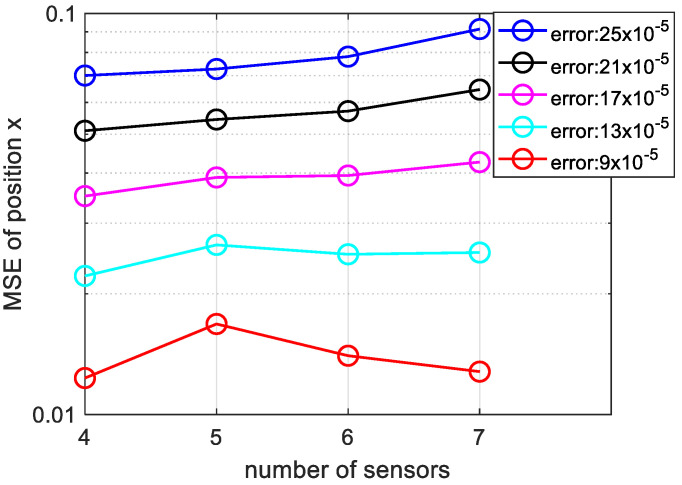
Variation of MSE of position x versus the number of sensors and varying measurement noise under bellhop underwater channel model.

Then we use the bellhop model to reproduce Figure 4, Figure 7 and Figure 8 as follows. As shown in Figure 9, even under the bellhop underwater acoustic model, the performance of the algorithm proposed in this paper is still better than the traditional TDOA positioning algorithm. The unit of abscissa in Figure 9 is $5\times 10^{-5}s,\;7\times 10^{-5}s$ and so on. Moreover, it can be inferred from Figure 10 that the algorithm proposed in this paper is suitable for underwater 3D localization, and its positioning performance is better than USR-LS. From the red and cyan lines in Figure 11, it can be seen that the positioning performance trends to improve with the increase of the number of sensors. The unit of error is seconds. However, when the noise is relatively large, a small increase in the number of sensors does not improve the performance of the algorithm according to purple, black and blue line in Figure 11. This is because noise error plays a major role. But if we increase the number of sensors, the positioning performance will be improved, as shown by the red line in Figure 11.

## 7. Conclusions

In this paper, we studied the problem of acoustic source localization in the underwater environment. We have investigated many kinds of literature about underwater acoustic sensor networks, positioning, and optimization algorithms. Regarding the particularity of the underwater environment, most positioning algorithms are used directly under the water, which causes a relatively large positioning error. Therefore, this paper firstly uses the TDOA algorithm and then uses the MM algorithm to optimize the obtained results to achieve the purpose of improving location accuracy. We also derived the SPEB’s mathematical expression for positioning problems that give the best expectations of unbiased estimation. The proposed T-MM algorithm has also been verified by simulation compared to the SPEB. The proposed T-MM is suitable for short-range scenarios, so we can assume that the propagation of the acoustic signal is a line of sight communication. However, in the actual situation, the propagation of acoustic signals underwater is a curve. So, to deal with that, in the future we plan to study the correction of sound rays based on the T-MM algorithm to improve the localization accuracy in field measurements. Simulation experiments were carefully conducted to test our proposed T-MM localization scheme using a statistical underwater acoustic channel model as well as the bellhop underwater channel model simulated channels. Both the statistical underwater acoustic channel model and the bellhop underwater channel model simulation results show that the proposed T-MM has better localization accuracy than the conventional acoustic-based underwater localization schemes.

## Figures and Tables

**Table 1:** Mean square error of all localization algorithms.

$\delta^{2}$	T-MM	SR-LS	USR-LS	SWLS	SDR	SPEB
$4.84\times 10^{-4}$	$5.18\;\times \;10^{-4}$	$7.01\;\times \;10^{-4}$	$8.62\;\times \;10^{-4}$	$1.70\;\times \;10^{-3}$	$1.23\;\times \;10^{-2}$	$4.44\;\times \;10^{-4}$
$4.84\times 10^{-3}$	$4.50\;\times \;10^{-3}$	$5.70\;\times \;10^{-3}$	$6.90\;\times \;10^{-3}$	$2.08\;\times \;10^{-2}$	$1.20\;\times \;10^{-1}$	$4.40\;\times \;10^{-3}$
$4.84\times 10^{-2}$	$5.19\;\times \;10^{-2}$	$6.53\;\times \;10^{-2}$	$8.30\;\times \;10^{-2}$	$1.61\;\times \;10^{-1}$	2.34	$4.44\;\times \;10^{-2}$
$4.84\times 10^{-1}$	$4.99\;\times \;10^{-1}$	$6.12\;\times \;10^{-1}$	$8.10\;\times \;10^{-1}$	3.95	44.98	$4.44\;\times \;10^{-1}$
4.84	4.94	6.42	7.93	75.30	115.09	4.44

**Table 2:** The water environment parameter of the Bellhop model.

Environmental Parameters	Water Column	Bottom Halfspace
Depth range	0~20 m	20 m
Compressional sound speed	1540~1543 ( m/s)	2000 m/s
Density	1021 kg/$m^{3}$	1810 kg/$m^{3}$
Shear sound speed	0	0
Compressional wave absorption	[6.93 × $10^{-5}$ 6.93 × $10^{-5}$] dB/km	0.5 dB/m
Shear wave absorption	0	0
Wind speed	5	
